# Designing a complex intervention for dementia case management in primary care

**DOI:** 10.1186/1471-2296-14-101

**Published:** 2013-07-17

**Authors:** Amy Waugh, Allana Austin, Jill Manthorpe, Chris Fox, Barbara Stephens, Louise Robinson, Steve Iliffe

**Affiliations:** 1 Mental Health Sciences Unit, University College London, Charles Bell House, 67-73 Riding House Street, London W1W 7EJ, UK; 2 Social Care Workforce Research Unit, King’s College London, London, UK; 3 Norwich Medical School, University of East Anglia, Norwich Research Park, Norwich NR4 7TJ, UK; 4 Dementia UK, 6 Camden High Street, London NW1 0JH, UK; 5 Institute of Health and Society, Newcastle University, Baddiley Clark Building, Richardson Road, Newcastle upon Tyne NE2 4AA, UK; 6 Department of Primary Care & Population Health, University College London, Royal Free Campus, Rowland Hill St., London NW3 2PF, UK

**Keywords:** Case management, Dementia, Research co-design, Primary care

## Abstract

**Background:**

Community-based support will become increasingly important for people with dementia, but currently services are fragmented and the quality of care is variable. Case management is a popular approach to care co-ordination, but evidence to date on its effectiveness in dementia has been equivocal. Case management interventions need to be designed to overcome obstacles to care co-ordination and maximise benefit. A successful case management methodology was adapted from the United States (US) version for use in English primary care, with a view to a definitive trial. Medical Research Council guidance on the development of complex interventions was implemented in the adaptation process, to capture the skill sets, person characteristics and learning needs of primary care based case managers.

**Methods:**

Co-design of the case manager role in a single NHS provider organisation, with external peer review by professionals and carers, in an iterative technology development process.

**Results:**

The generic skills and personal attributes were described for practice nurses taking up the case manager role in their workplaces, and for social workers seconded to general practice teams, together with a method of assessing their learning needs. A manual of information material for people with dementia and their family carers was also created using the US intervention as its source.

**Conclusions:**

Co-design produces rich products that have face validity and map onto the complexities of dementia and of health and care services. The feasibility of the case manager role, as described and defined by this process, needs evaluation in ‘real life’ settings.

## Background

Dementia is one of the main causes of disability in later life. One in 14 people aged over 65 years has a form of dementia, rising to one in six of those aged over 85. In the UK, currently around 700,000 people have dementia but this is estimated to rise to 1 million by 2020 and 1.7 million by 2050, an increase of over 150% [1]. The current costs of caring for people with dementia in the UK have been estimated at between £17 and £18 billion a year. Currently around two-thirds of people with dementia live at home, with the majority of their care provided by family members with support from primary and social care teams.

The decline in the number of long-term care home places, together with the rising numbers of older people, will lead to increasing numbers requiring complex care packages if they are to continue to live in their own homes and postpone or avoid moving into care homes. The policy imperatives are clear. In England, improving the health and social care of our ageing population is a key policy priority [2,3]. The White Paper *Our health, our care, our say*, stipulated that care for older people should be delivered as close to their homes as possible [4]. NICE-SCIE (2006) guidelines recommend that co-ordinated care led by a single professional should be provided for people with dementia.

However, the growing numbers of people with dementia, many of whom have other long-term health conditions, present considerable challenges for primary care. There is evidence that the standard of dementia care in the UK is in urgent need of improvement, with frequent failure to deliver services in a timely, integrated or cost-effective manner [5,6]. Within primary care, General Practitioners (GPs) admit to difficulties both in dementia diagnosis and common areas of dementia management [5]. In the UK, dementia detection rates have been increased through the use of educational interventions in primary care but these interventions did not have an effect on clinical management [7].

International research has revealed the potential benefits of a collaborative, case management approach to the assessment [8] and care of people with dementia [9,10]. In the United States (US), the PREVENT [9] study showed benefits when case managers used care pathways and evidence-based protocols to manage neuropsychiatric symptoms encountered by family carers during the dementia disease trajectory. It demonstrated significant improvements for both people with dementia (increased prescribing of cholinesterase medication, fewer behavioural and psychological symptoms) and for their family carers (higher carer satisfaction ratings); however due to a limited follow-up period, effects on rate of moves to long-term care facilities and cost-effectiveness could not be determined.

Following the recommendations for primary care services from the World Alzheimer Report [11], testing a case management approach to people with dementia in NHS primary care looks attractive, but there are grounds for caution. A critical review of nurse-led case management as a technique for supporting patients with complex needs warns against expecting substantial benefits from the case management approach [12]. A French team recently published a systematic review of randomized controlled trials of case management for people with dementia and their caregivers, with time-to-institutionalisation and cost as the main outcome variables. They concluded that the evidence for the efficacy of case management in terms of cost and resource usage remains equivocal, and that further studies ought to consider which individuals might benefit particularly from case management [13].

In response to this review, we argued that a detailed specification of the sorts of activities to be included in case management was needed, with an understanding of how case managers might tailor their support to the needs of the person with dementia and their family [14]. What remains to be learned, in our view, includes: (1) determining which skills are most appropriate to the role; (2) where these may be located; (3) which cohort of patients with dementia would benefit most from case management; and (4) the type and intensity of contact. To explore these issues we are undertaking the CARE-DEM study [15].

**Figure 1:**
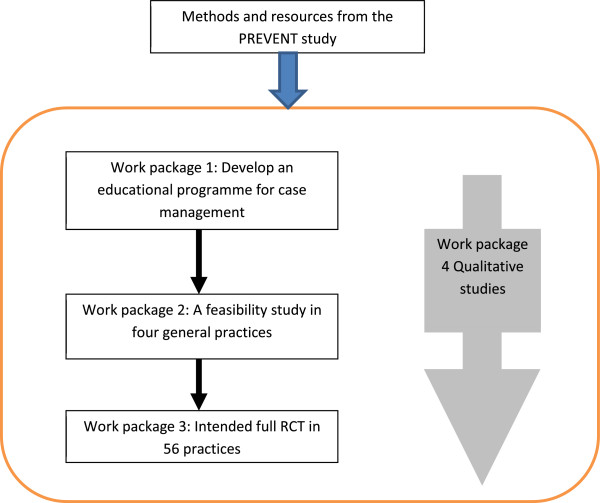
Diagram showing the overview of the full planned CARE-DEM research project.

The CARE-DEM study is a research and development project aimed at translating and adapting the PREVENT intervention to, and testing it in, English contexts. This could allow the optimal design of a definitive randomised controlled trial to evaluate feasibility, acceptability, cost and clinical effectiveness (for an overview of the full planned CARE-DEM research project see Figure 1) . The aim of this paper is to report and discuss the adaptation of the PREVENT intervention to the setting of the English National Health Service (NHS) and local government Adult Services, by practitioners and people with experiences of dementia services (including carers and members of third sector organisations).

## Methods

This study was grounded in the recommendations of the Medical Research Council’s guidance on complex interventions which asserts: *‘Best practice is to develop interventions systematically, using the best available evidence and appropriate theory, then to test them using a carefully phased approach, starting with a series of pilot studies targeted at each of the key uncertainties in the design, and moving on to an exploratory and then a definitive evaluation.’*[16].

Given the uncertainties surrounding case management for dementia, we approached the adaptation of the PREVENT intervention as a design process for an innovative way of working. The adoption of new ways of working may depend on the characteristics of the new approaches themselves, and those of the professionals and patients/carers who use them [17]. The characteristics of innovations that favour their uptake and diffusion through clinical practice [18-20] are: their compatibility with the values, norms and perceived needs of intended adopters; their simplicity; the clear, unambiguous advantage they offer; their openness to experimentation by intended users; the ability to adapt, refine or otherwise modify the innovation; their ability to add to the user's social standing; and the voluntary nature of their use. We were conscious that attempts to introduce new methods of working and new technologies into primary care are often unsuccessful. There is a risk of ‘shoe-horning’ – forcing the innovation into practice – that provokes resistance to change amongst practitioners [21].

We assumed that a complex process of case management for a complex disease, namely dementia, would work against standardisation of clinical methods and in favour of professional creativity and person-centredness. *“Standardising care without identifying desirable variation or unique adaptations that take advantage of local opportunities or strengths misses an opportunity to identify and investigate unanticipated circumstances or locally adapted practice configurations associated with better health care outcomes”*[22]*.*

Full ethical approval was granted from the North West London Research and Ethics Committee (10/H0722/50) and all work was carried out within the guidelines set by the declaration of Helsinki.

### The design process

The aims of work package one (WP1) of the CARE-DEM study were to review, adapt and customise the PREVENT intervention for NHS general practice. WP1 corresponds to the first stage (development) of Mohler et al.’s criteria for describing complex interventions [23].

We used a co-design method to gain insight from a diverse range of experienced practitioners and carers [24]. Following meetings of the co-design development group, the materials produced were then reviewed and critiqued by a separate group of practitioners, carers and older people with experience of using health and social care services. This was a cyclic process in which a series of prototypes was refined until the development group felt confident that it had produced a version worth field-testing in work package two (WP2).

Prior to each meeting, the materials for discussion and any amendments that had been made from the previous meeting were circulated via email to members. Those unable to make a meeting were invited to email their comments in advance. This process was repeated with each of the outputs until the group declared itself happy with the materials in a final review meeting.

We anticipated that contextual issues underpinning the development process would be:

1) The staff likely to become case managers would be experienced NHS or social work professionals, so principles of adult learning would core to the intervention;

2) Case managers would need to be flexible in working with geographical variations in service and professional availability, and the differing boundaries of organisations;

3) They would need to navigate through changes underway in health and social care services.

### Setting and process

The research team undertook development work in one area of England to facilitate group meetings. The site chosen covered a diverse population and different organisational boundaries. Research staff in Kent and Medway NHS and Social Care Partnership Trust were asked to identify potential members for an expert group of stakeholders, and to recruit a range of people and professionals, including family carers as well as health and social care practitioners. It was not possible to identify a person with dementia to join the group and only one family carer was recruited. Individuals were invited to a preparatory meeting to explain the study and to outline what commitment was being requested (such as attendance at six meetings). Refreshments and travel costs were met by the research grant and ‘back-fill’ money was available to meet the cost of attendance of NHS professionals. Venue costs were met by the research grant. Ethical permission for this part of the study was required and successfully sought (NW London Rec1 10/H0722/50) and local research governance permissions were obtained.

### Development group

Twelve people volunteered to join the core multidisciplinary group which consisted of an occupational therapist, social workers, an Admiral nurse (community nurse specialising in dementia support), a family carer, a consultant psychiatrist, a GP, a community psychiatric nurse, and an outreach worker from the local branch of the Alzheimer’s Society. Practical arrangements, including meeting room arrangements, refreshments, invitations, circulation of documents, and so on, were undertaken by local research support staff.

The group met six times from April 2010 to June 2011, using a nominal group technique to carry out the following tasks:

1) To adapt the PREVENT intervention to meet service and cultural expectations in England;

2) To devise a job description and a list of desirable and essential attributes for a case manager;

3) To agree the contents of an educational needs assessment that would inform training and mentoring;

4) To produce written information designed to be used by the case manager with carers and people with dementia.

Nominal groups are a potentially powerful learning and development tool [25] which have a particularly useful role in analysing health care problems [26], and can help bridge the gap between researchers and practitioners [27]. A nominal group technique was used, rather than a focus group, to encourage participants to solve specific problems (e.g. how to revise the PREVENT approach to fit with English care systems and nomenclature) rather than to explore the subject generally.

A nominal group technique designed for complex problems enabled this small group to debate the subject specifics and to contribute from their own experiences. This required the group to generate ideas, confirm that they were addressing the same problem, analyse the content of the ideas, categorise ideas and clarify the items in each category [28]. The nominal group meetings were led by a member of the research team who facilitated discussion to cover the key questions, and focused attention on achieving a common understanding of these questions and their answers, whilst two other researchers, acting as participant observers, took notes.

### Review group

A wider group of professionals and carers furthered the co-design principles by offering their comments on the work of the development group. The research team recruited ten professionals from different parts of England to provide comment in writing by email and arranged a meeting of 11 older people with substantial experiences of using health and social care services, including current and former carers of people with dementia. This was a diverse group, including people from different ethnic backgrounds, different sexual orientations, different socio-demographic characteristics, and holding a range of family or caregiving relationships (such as spouse/partner carers and adult child carers). Some were members of voluntary sector groups, such as the Alzheimer’s Society, others had connections with older people’s or community based organisations. The group membership was drawn from different locations than the nominal group in order to reflect a broader range of current service arrangements. A presentation was given on the objectives and outputs of WP1, specific questions were asked of the group and they were asked for their views. This group provided feedback through intense and detailed group discussion, they were also encouraged to contact the team via email if they had anything further to add after the meeting. This group served as a helpful validation step, in their constructive comments which helped to provide some assurance that the products of the development group were transferable to other parts of England.

## Results

The development group created a case manager job description, a person specification and an educational needs assessment to assist recruitment, induction to the role and further training. A case management ‘manual’ was also created (modified from the PREVENT study). This manual also included accessible leaflets for people with dementia and their carers, which could be used as an opportunity for information-giving and as talking points between case managers and their clients.

### Job description and Person specification

The development group was mindful during the development of the job description and person specification that the role it was developing should not overlap with existing roles. The group discussed which professionals might be best suited to this role and it was agreed that nurses would be the most obvious choice (as in the PREVENT trial), but that other allied health and social care professionals might also be suitable. The job description therefore focused on skills rather than possession of professional qualifications. The group were mindful that some professionals, for example doctors, were expensive and therefore unaffordable. Important themes raised in the discussions included:

• **Interaction:** Case managers would benefit from communicating with each other regularly to share knowledge and experiences.

• **Mapping resources:** The role would require practitioners to be proactive and ready to identify current resources to support people with dementia and their carers, whilst identifying gaps in provision.

• **Overload risks:** There was a risk that the role might overwhelm the case manager and that working relationships with specialist teams might reduce this risk. It was agreed that the feasibility study (WP2) needed to highlight any overload or stressors.

The job description for the dementia case manager role is shown in Table 1 and the person specification in Table 2.

**Table 1:** Job description of a primary care case manager

**Case management is the provision of coordinated health and social care by a single health or social care professional**	
Case managers in the CARE-DEM trial will be employed by NHS organisations, and will work under the supervision of a GP or other clinical lead, and an NHS line manager.	
The case manager in the CARE-DEM trial will undertake the following tasks:	
1.	Identify people with dementia (PWD) from general practice lists.
2.	Review medical records of PWD +/- their carer(s), noting any gaps in the record and also the involvement of other possible sources of support.
3.	Liaise with other professionals who know the PWD to learn their perspectives on individual or family needs.
4.	Engage with the PWD +/- carer to identify their main concerns or unmet needs.
5.	Update or fill in gaps in GP medical records and where appropriate update social care records.
6.	Analyse information obtained with PWD & carers.
7.	Map support available to and wanted by PWD & carer. Create a personal care or support plan with each PWD & carer, and initiate actions that will provide that support (for example, help with seeking advice about benefits, liaising with the GP about treatment of other conditions and discussion of plans around finance, health and welfare decisions^1^)
8.	Analyse information obtained with other relevant practitioners (e.g. GP, social worker, care home key worker).
9.	Prioritise individual PWD and carers: Assess need for action in terms of ‘intensive’, ‘maintenance’ and ‘holding’ (for those already being case managed by other agencies).
10.	Build the care plan into the GP medical records, and share with other disciplines and agencies as needed
11.	Organise systematic follow-up to review the outcomes of actions taken, meet regularly with the GP or other relevant clinical leads, and act as an advocate for the PWD and carers.
12.	Meet regularly with his/her mentor, to discuss PWD and carers with whom they are working, to review prioritisation, to resolve any problems that have arisen and to plan the end of their role with the PWD and their carers, as appropriate.
13.	Undertake professional updating and top-up training, as needed.
14.	Meet with and communicate with members of the research team to discuss the case manager role as it develops.

^1^Details inserted here to limit the scope for interpretation of the job by those doing it.

**Table 2:** Skill set for a dementia case manager in primary care

**Attribute/skill**	**Desirable or Essential**
Hold a relevant qualification for their discipline	E
Basic IT skills, knowledge of local IT systems and experience in recording information electronically	E
Interpretation of medical and nursing records	E
Communication skills, particularly with difficult topics (diagnosis itself, prognosis, BPSD, continence, anxiety)	E
Person-centred (respects autonomy), non-judgemental attitudes and values	E
Awareness of confidentiality, family dynamics, adult safeguarding, sensitivity of financial issues, taboos (e.g. continence)	E
Skilled in maintaining dialogues, shared decision-making, interagency communication, ability to seek agreements on data sharing	E
Experience in decision making, risk assessment, prioritisation	E
Verbal and written communication skills, ability to negotiate, able to create relationships and respect boundaries	E
Openness to learning, prepared to develop skills	E
Good at managing tensions and contradictory demands, good time and stress management skills	E
Already working in local NHS or adult services	D (could be a returner etc)
Knowledge of local dementia and older people’s & carers’ services	D
Capable of system-building, networking and increasing efficiency within services	D
Skills in empowering PWD & carers to identify and solve problems	D
Able to vary involvement according to PWD’s and carers’ needs	D

Applicants for the case-manager roles in CARE-DEM should have the following attributes and skills.

### Educational needs assessment

Educational needs assessment [29] was developed during the early years of the Evidence-Based Medicine movement where it was used by Sackett and colleagues to mobilise knowledge and evidence in clinical settings [30]. The needs assessment approach derives from adult learning theory as applied to clinical practice [31]. During the development of the educational needs assessment, the group discussed case management tasks, competencies required or desirable for them, risks to minimise, and the tools required to undertake the case management role successfully. These conversations resulted in the production of a task matrix (see Table 3). This matrix is work in progress, in that the development group did not complete each cell, expecting that some further risks and tools would emerge in the feasibility study (WP2).

**Table 3:** Educational needs assessment matrix

**Tasks**	**Competencies required**	**Risks to avoid**	**Tools required**
Identify PWD from practice list	Knowledge of local IT systems or links with practice staff with such knowledge		
Review medical records of PWD +/- carer, noting any gaps and involvement of other possible support systems	Interpretation of medical and nursing records, and knowledge of local dementia services		Checklist or data extraction form
Liaise with other professionals who know the PWD to learn their perspectives on individual or family needs	Knowledge of local services and agencies	Accept professional assumptions about PWD and carer needs, too readily	
Engage with PWD +/- carer to identify their main concerns or unmet needs , update or fill in gaps in medical records	Communication skills, particularly with difficult topics (diagnosis itself, prognosis, BPSD, continence, anxiety)	Stigmatisation	Semi-structured conversation schedule?
	Person-centred (respects autonomy), non-judgemental	Duplicating assessments	Accurate information
		Triggering fears (inspection, judgement, loss of control, interference)	
Mapping support available to and wanted by PWD & carer. Analyse information obtained with PWD & carer,	Analysis and recording, and knowledge of local resources. System-building, increasing efficiency	Antagonising existing carers and support workers	Matrix of available support and current needs
	Awareness of confidentiality, family dynamics, adult safeguarding, sensitivity of financial issues, taboos (continence)	Just signposting – must act and do	
Analyse information obtained with other relevant practitioners (e.g.GP, social worker, care home key worker)	Dialogue, shared decision-making, interagency communication, ability to seek agreements on data sharing		
	Function as ‘connective tissue’		
Prioritisation: assess need for action in terms of ‘intensive’, ‘maintenance’ and ‘holding’(for those already being case managed by other agencies)	Decision making, recording skills, risk assessment, prioritisation	Create work for others	Definitions of intensive, maintenance & holding
	Promoting problem-solving by PWD & carers		
	Health maintenance & promotion skills		
Create a personal care or support plan with PWD & carer, and initiate actions (see JD for examples)	Problem-solving approach	Promising more than can be delivered	Care plan proforma? Agreed by all stakeholders?
	Verbal and written communication skills, negotiation		
Organise systematic follow-up to review outcomes of actions	Organisational skills, use of electronic reminder systems	Duplicating others’ work, not fitting into current local plans for service changes	
	Tapering down involvement if needs reduced, stepping up when necessary		

The overarching topics considered most important by the group were: how existing competencies of case managers should be assessed in meeting the emotional needs of a person with dementia and their carers, and how to develop the skills of the case manager in areas where these could be improved. This was seen as important as each case manager was likely to bring different experiences and attributes and an adult learning approach would build on these and not assume that a common training package would suit all.

The competencies, risks and tools identified in the task matrix were used as the basis for an educational needs assessment tool. This mapped competencies onto the dementia disease trajectory, under five headings:

1) People who are acquiring or who have just received a dementia diagnosis.

2) Managing breakdown of support systems.

3) Managing acute illness and hospital admission.

4) Supporting decisions about relocation.

5) Supporting the person with dementia at the end of life and their family.

Sub-headings were agreed for each of the five main headings (see Table 4). This educational needs assessment was designed for use in the induction process for the case manager, but also as a topic guide for mentoring during active case management.

**Table 4:** Educational needs assessment for CARE-DEM case manager’s learning, induction and refresher courses

**Thinking about…**	**Themes**	**Confident about this**	**Need to learn about this**
1. People who are acquiring or who have just received a dementia diagnosis	Able to establish relationship with the individuals & their family that is at levels and intensity of protocol		
	Informed about sources of support locally (and beyond), including peer support		
	Able to inform practice with knowledge of memory aids & techniques		
	Able to reframe dementia as a disability		
	Able to assessing individual/family adjustment to and assimilation of the diagnosis, able to set assessments in interprofessional and multi-agency frameworks		
	Able to reinforce resilience		
	Aware of how to introduce advance care planning and other possible planning/decisions		
	Aware of psychosocial interventions and their availability, effectiveness and cost		
2. Managing breakdown of support systems	Able to analyse and respond to behavioural & psychological symptoms		
	Able to support person/carer to access sources of support for crisis and ensure these are as effective as possible		
	Able to identify and analyse support networks and to develop or sustain support		
	Know how to advise about incontinence/ aids and		
	equipment/safeguarding/ housing/community based social care and other opportunities		
3. Managing acute illness and hospital admission	Able to command confidence and exhibit negotiation skills in liaison with multi-disciplinary team. Able to advocate on the person’s behalf or support them in self-advocacy. Able to advise on re-ablement.		
4. Supporting decisions about relocation	Aware of resources and implications of relocation and able to discuss them with the individual to assist in considered decision making		
5. Supporting the PWD and their family at the end of life	Able to command confidence that support will be available and that decision making will be personalised. Able to elicit fears and concerns about management of crisis, distress and pain. Able to offer support to bereaved carers and other members of the support network.		

Mentoring was seen as essential to the introduction of case management approaches in primary care, since the new case managers would be learning through the experience of working with a diverse patient group.

### The manual

The manual focused on ways in which a case manager would work with a family carer around the following topics: communication with the person with dementia, behaviour problems, mobility, personal care, sleep, legal and financial issues, physical health, depression and anxiety, and how to respond to psychotic symptoms.

A number of rules were developed in the adaptation of the PREVENT manual, to systematically alter the language and tone of the US version. These included:

• Removal of all references to the person with dementia as the ‘loved one’ and replacement of this term with ‘relative’.

• Use of words like ‘try’, ‘consider’ and ‘may’ to make the manual less directive and prescriptive, and deletion of phrases like ‘instruct the carer’.

• Replacement of phrases with a negative tone (advising carers not to do things) with more positive actions or things to try. Here the group added explanations for why the person with dementia might behave in a certain way and tried to make the manual more person-centred by explaining that symptoms and difficulties were likely to vary from time to time and from person to person.

Information about local NHS and the social care services, and about the Alzheimer’s Society and local support organisations, was added to the manual. Suggestions that the carer should speak to the case manager were included to make the manual more interactive, ‘Key points’ boxes and subheadings were added and the order of contents was re-arranged to provide a more coherent structure. Images were removed where these were inappropriate for the English context and distracted from the content. The development group decided to add an introduction and contents page. Pages on asking for help, looking after yourself, physical health, aggression and agitation, depression and anxiety, and planning for the future were also added since the group members thought these were important and could be over-looked unless specifically considered.

### A page from the manual

#### ***Communication***

Everyone is different and your relative will have their own way of communicating with you. This could change over time and become challenging or frustrating. There are ways you can make communication easier and these may also help maintain your relative’s independence.

#### ***Your Communication***

• Listen carefully.

• Use a calm, gentle manner with open body language e.g. not crossing your arms.

• Speak slowly and clearly to ensure your relative can hear you.

• Use positive facial expressions such as smiling or nodding.

• Use short, familiar words and simple sentences.

• You may find that you need to repeat yourself more frequently, this can be frustrating. Try to remember it is not their fault, it is a result of memory loss.

• Give one direction or ask one question at a time.

➢allow plenty of time for your relative to respond.

➢if there is no response, repeat exactly what you said.

• It may sometimes be necessary to remind your relative who you are.

• If your relative has difficulty finding the right words.

➢prompt them to find the word, by asking questions or providing visual cues (e.g. showing a cup).

➢if they are still having difficulty, try guessing what they mean and check you have guessed correctly.

• If this becomes difficult, take a break and try again later.

#### ***Attention***

• Talk in a quiet place and try to reduce distractions e.g. turn down the TV or radio.

• Physical contact can help to get your relative’s attention and reassure them (e.g. touching them on the arm).

• Make sure your relative can see you clearly e.g. have good light in the room and position yourself so you are facing them.

• Making eye contact at the start of a conversation can show your relative you are talking to them.

• It helps to be in the same room and not shout from other rooms.

#### ***Visual Prompts and Reminders***

• It can be helpful to use large, clear calendars, diaries or clocks.

• To help your relative identify where items are it may be helpful to have pictures or labels e.g. pictures of plates on the kitchen cupboard or important telephone numbers by the phone.

• Describing the steps of a task or activity with your relative can be useful e.g. when helping with dressing describe each step in advance.

• Miming actions can support what you are saying e.g. when asking them to brush their teeth you could also mime the action.

➢Always treat your relative with dignity and respect.

➢Try not to take negative things they say too personally.

➢Communication can be difficult, sometimes it is better to end a conversation and come back when you are both calmer.

## Discussion

The co-design approach used in this project led to the successfully to the development of a skill set for a primary care dementia case manager, a method for educational needs assessment, and a process of induction and mentoring to allow the case manager role to be adopted by existing primary and social care staff. The skill set focused on the establishment and maintenance of trusting and respectful working relationships between the case manager, the person with dementia, their family carers and other professionals involved in their care and support. These principles match those promoted by the English Department of Health in its guide to training the social care and health workforce [32], but the skill set differs in its use of the dementia disease trajectory as an organising principle applying case management competencies. Educational needs assessments originated as a way of integrating evidence in the clinical care of patients, but we used a modified approach [33] to create a diagnostic tool that can identify the topics or tasks about which the case manager needs to learn rapidly. The induction and mentoring process envisaged in the deployment of primary care-based case managers emphasises the social interaction between novice case manager and experienced mentor typical of ‘situated learning’, learning that occurs within communities of practice [34].

There is much that can be learned from WP1 and the account of this developmental stage of a complex intervention. For example, it was possible to obtain the sustained engagement by frontline practitioners and carers in designing the intervention. Group dynamics, within a multi-professional and multi-agency nominal group, were productive and good humoured. The views of family carers enriched discussion and materials produced took into account practitioners’ knowledge of the realities of local health and social care systems.

There may, however, be limitations of this development process: for example, the views of people with dementia may not have been given sufficient weight, some professional perspectives and opinions may have dominated the group, and the expert development group may have not used the review group’s comments sufficiently to guide re-developments of materials. Additionally most of the development group members worked within one NHS Trust and local authority and potentially lacked understanding of the different skills needed and the challenges of delivering this intervention in other settings where organisations are more or less integrated or differently structured.

During the process of developing the intervention materials and protocols, many concerns were raised by health and social care professionals and older people regarding the case manager’s role and the challenges of delivering this intervention. The main areas highlighted relevant to the role of a case manager were how to resist giving information as a simplistic solution to problems, how to avoid adding too many elements to the initial needs assessment and how to taper involvement as needs changed. Other difficulties discussed were how to reduce fragmentation of care through multiple referrals, the complexity of recording sensitive information, and working with risk and the possible abuse of older people. After much discussion the research team felt they understood these concerns and uncertainties and highlighted the investment needed in regular case manager supervision from a senior Admiral Nurse in the WP2 feasibility trial.

The development group also discussed the challenges of delivering this intervention in primary care. Group members voiced concerns that many GPs do not understand the impact of dementia and do not refer people on to support services and that this might affect the potential work of a case manager and their ability to engage with the wider primary care team. Most members were aware that associations of dementia are often very negative and that carers are often scared to seek and accept help, so that a case manager might be less frequently accessed than predicted. However, the development group unanimously considered that the trial of a case management system working in a model of collaborative care would be very timely with the growing interest and funding for dementia support within primary care in the UK, especially since Callahan and colleagues [33] have recently demonstrated how system-level barriers to implementing a complex intervention can be overcome.

The learning resources, workplace training methods and customised care pathways produced by the development group are now being tested in a feasibility trial. This involves a ‘field test’ of the intervention in four general practices, in three separate areas of England, to determine if it is both feasible to use in English NHS general practice, and acceptable to people with dementia, their families and other professionals. This may result in further amendments to any part of the intervention.

## Conclusions

Evidence to date on both the clinical and cost effectiveness of a case management model in dementia care has been equivocal; however following the publication of the 2011 World Alzheimer Report on primary care and early intervention service [11], interest in the implementation of such a model to improve care co-ordination is growing. This paper discusses the adaptation of a US dementia case management intervention for the English NHS, by practitioners and people with experiences of dementia services, including carers and members of third sector organisations.

There is increasing interest in the involvement of people with experiences of using health and care services in research at all levels [34-36]. This account of a primary care initiative has outlined the ways in which this can be done using the principles of co-design and co-working over a sustained period of one year. There is also a strong case for involving front-line professionals in the design of practice-near studies and this too can be sustained. The key ingredients for the success of this work package appeared to be a blend of local and national perspectives; close attention to the practicalities of meetings and communications, a task-focused approach and the engagement of practitioners from a range of disciplines and agencies that were keen to work together on developing support for people with dementia.

## Abbreviations

UK: United Kingdom; NICE-SCIE: The National Institute for Health and Clinical Excellence (NICE) and the Social Care Institute for Excellence (SCIE); GP’s: General practitioners; US: United States; NHS: National health service; WP1: Workpackage one; WP2: Workpackage two.

## Competing interests

The authors declare they have no conflicts of interest with the contents of this manuscript.

## Authors’ contributions

SI, JM, CF, LR and BS were involved in the project design and acquisition of funding. AA and AW were responsible for the project implementation and data capture. All authors were involved in the co-ordination and write up of the project. All authors read and approved the final manuscript.

## Pre-publication history

The pre-publication history for this paper can be accessed here:

http://www.biomedcentral.com/1471-2296/14/101/prepub
